# Supplementary material for: Ensemble forecasts of COVID-19 activity to support Australia’s pandemic response: 2020–22
Source: PLoS Comput Biol. 2026 Apr 22;22(4):e1014199. doi: 10.1371/journal.pcbi.1014199 (PMC13124057; doi:10.1371/journal.pcbi.1014199)
Supplement: S1 Text — Contains supporting figures. (PDF) [file pcbi.1014199.s001.pdf]

Ensemble forecasts of COVID-19 activity to support Australia's pandemic response: 2020–22

Robert Moss[1] Ruarai J. Tobin[1] Mitchell O'Hara-Wild[2]
Adeshina I. Adekunle[3] Dennis Liu[4] Tobin South[4] Dylan J. Morris[4]
Gerard E. Ryan[5,1] Tianxiao Hao[5,1] Aarathy Babu[5]
Katharine L. Senior[5.1] James G. Wood[7] Nick Golding[5,6]
Joshua V. Ross[8,9] Peter Dawson[3] Rob J. Hyndman[2] David J. Price[1,10,*]
James M. McCaw[1,11,*] Freya M. Shearer[1,5,*]

[1]: Melbourne School of Population and Global Health, The University of Melbourne; [2]: Department of Econometrics and Business Statistics, Monash University; [3]: Defence Science and Technology Group; [4]: School of Computer and Mathematical Sciences, The University of Adelaide; [5]: The Kids Research Institute Australia; [6]: School of Physics, Mathematics and Computing, University of Western Australia; [7]: School of Population Health, University of New South Wales; [8]: Department for Health and Wellbeing, Government of South Australia; [9]: South Australian Health and Medical Research Institute; [10]: Department of Infectious Diseases, The Peter Doherty Institute for Infection and Immunity; [11]: School of Mathematics and Statistics, The University of Melbourne; [*]: Equal authorship

Contents

1 Ensemble forecasts for each phase of the study period 2
2 Forecast evaluations around observed epidemic peaks 7
3 Model rankings for each ensemble forecast 11
4 Model skill scores for the Pre-Delta wave in Victoria 12
5 Probability integral transform (PIT) histograms 13
6 Marginal quantile and CDF calibration plots 15
7 CRPS values for each dominant strain 17

1 Ensemble forecasts for each phase of the study period

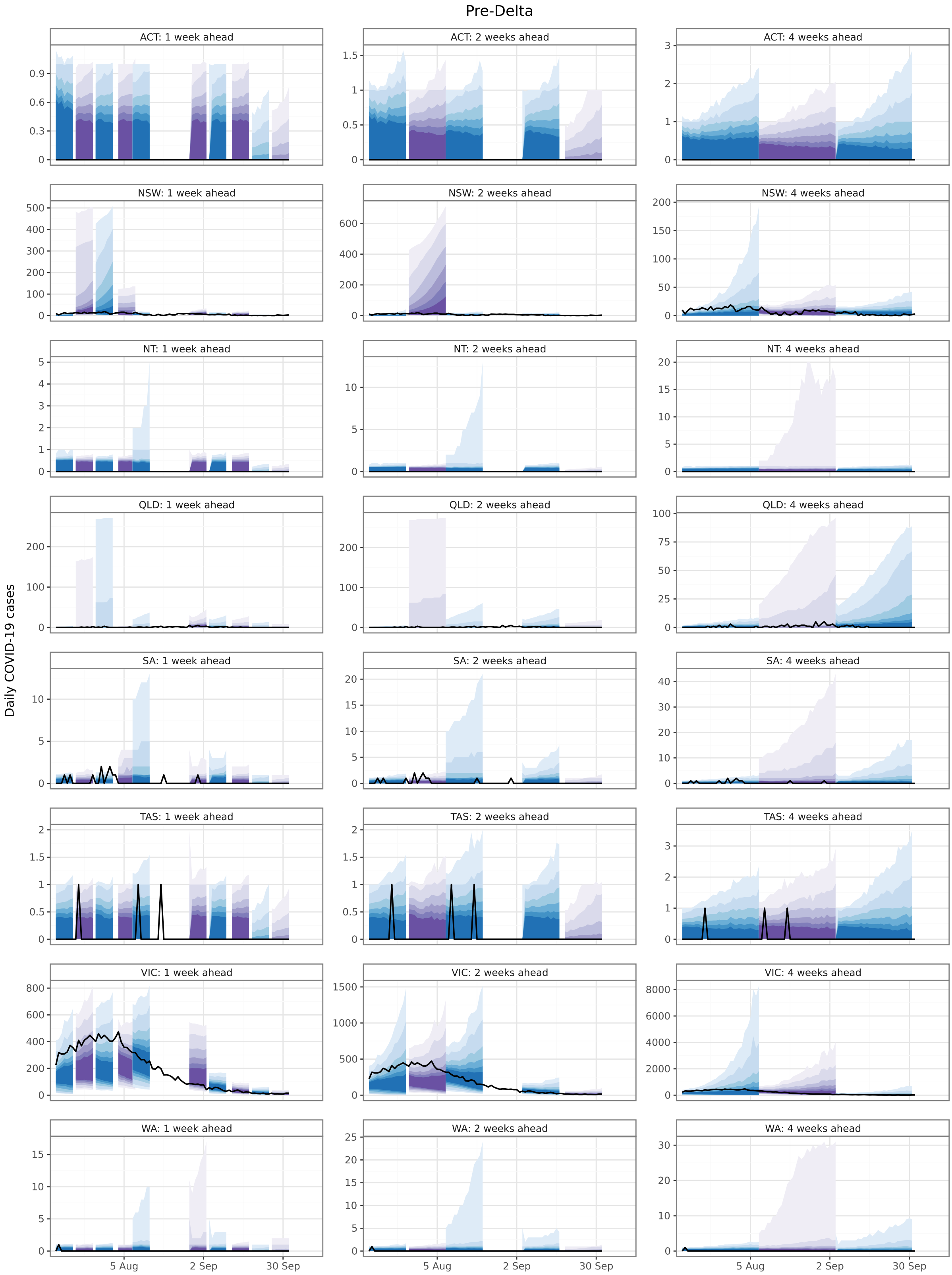

Figure A: Ensemble forecasts for the "Pre-Delta" period (2020).

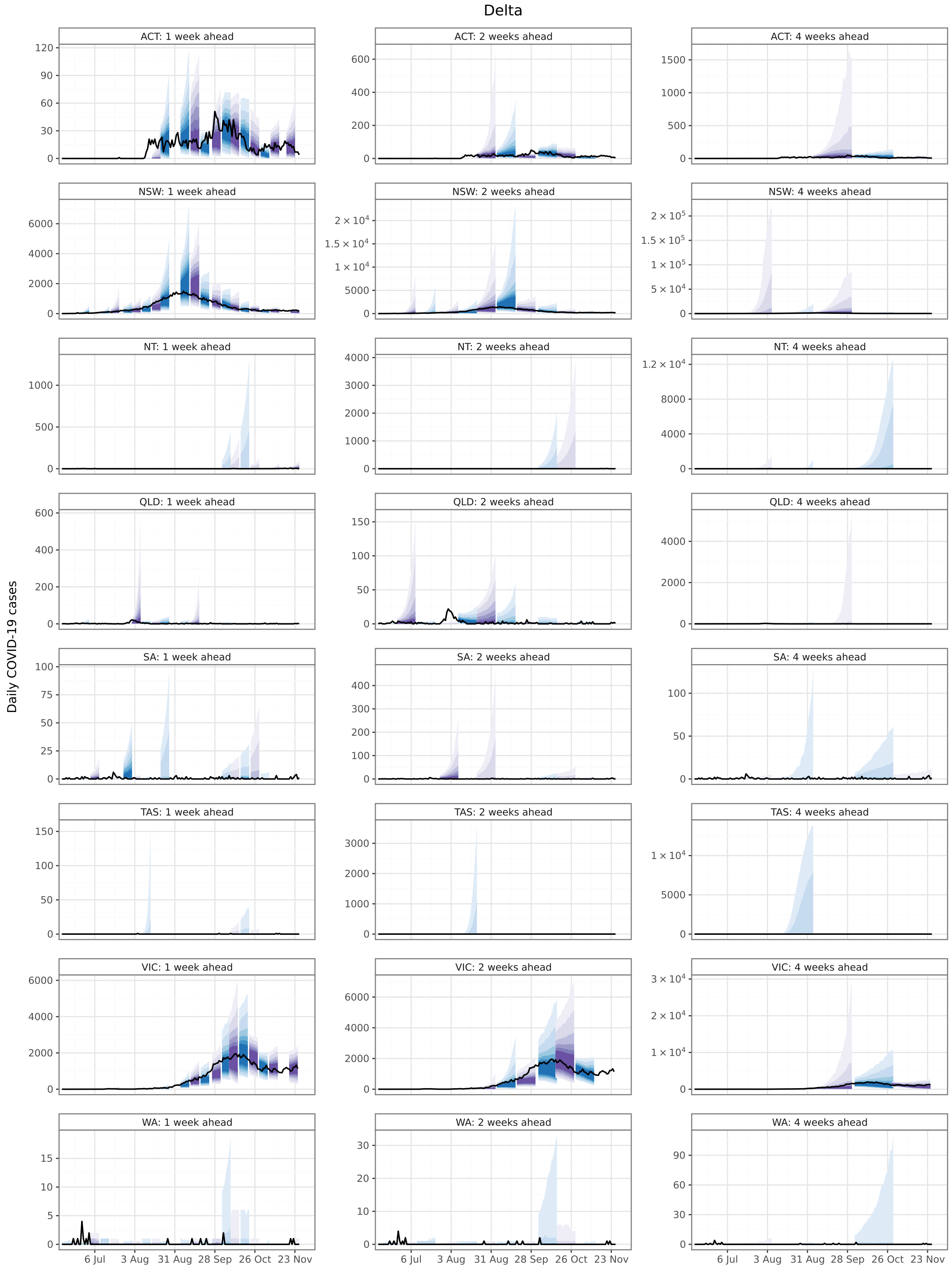

Figure B: Ensemble forecasts for the "Delta" period (2021).

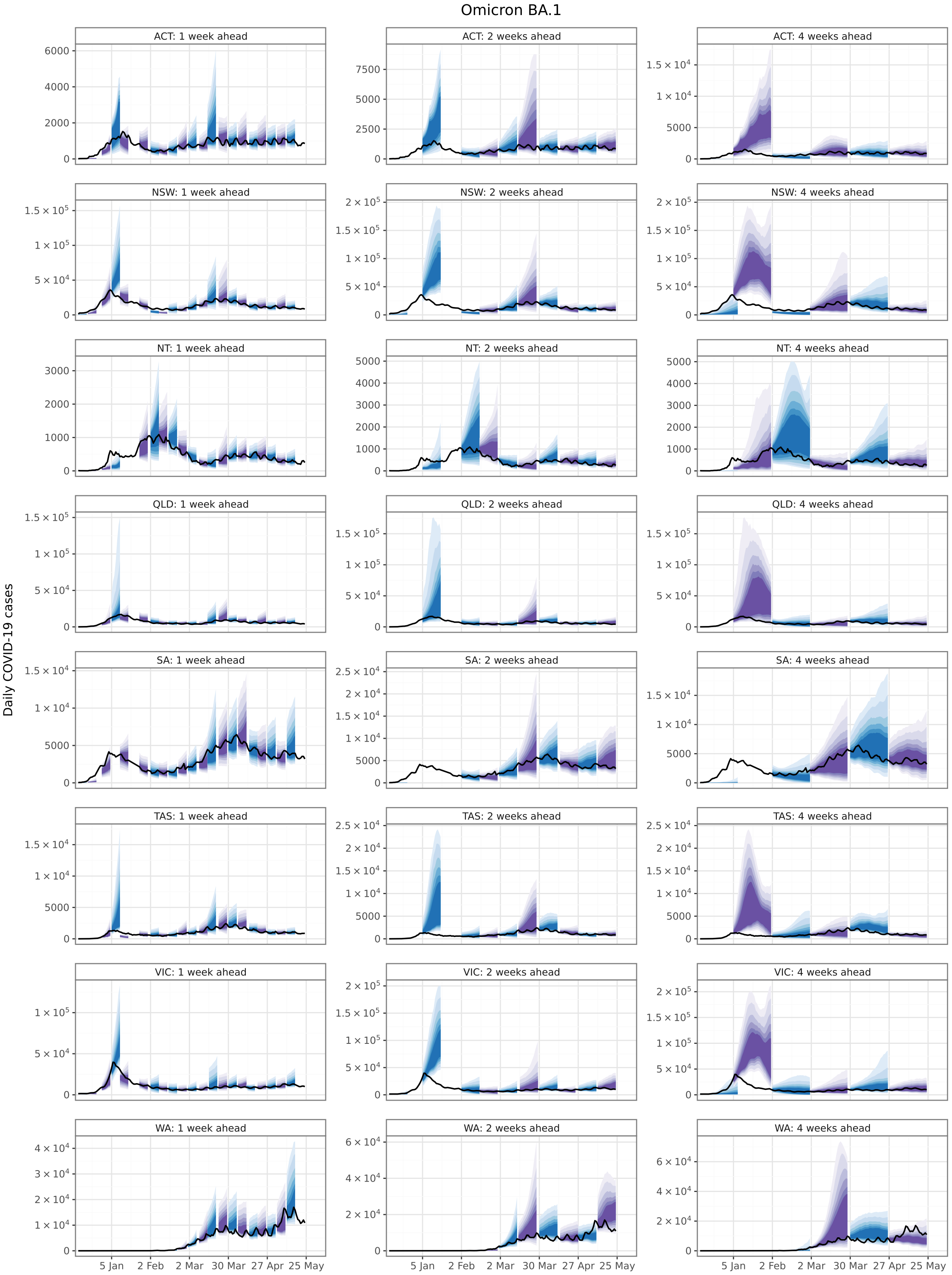

Figure C: Ensemble forecasts for the “Omicron BA.1” period (2022).

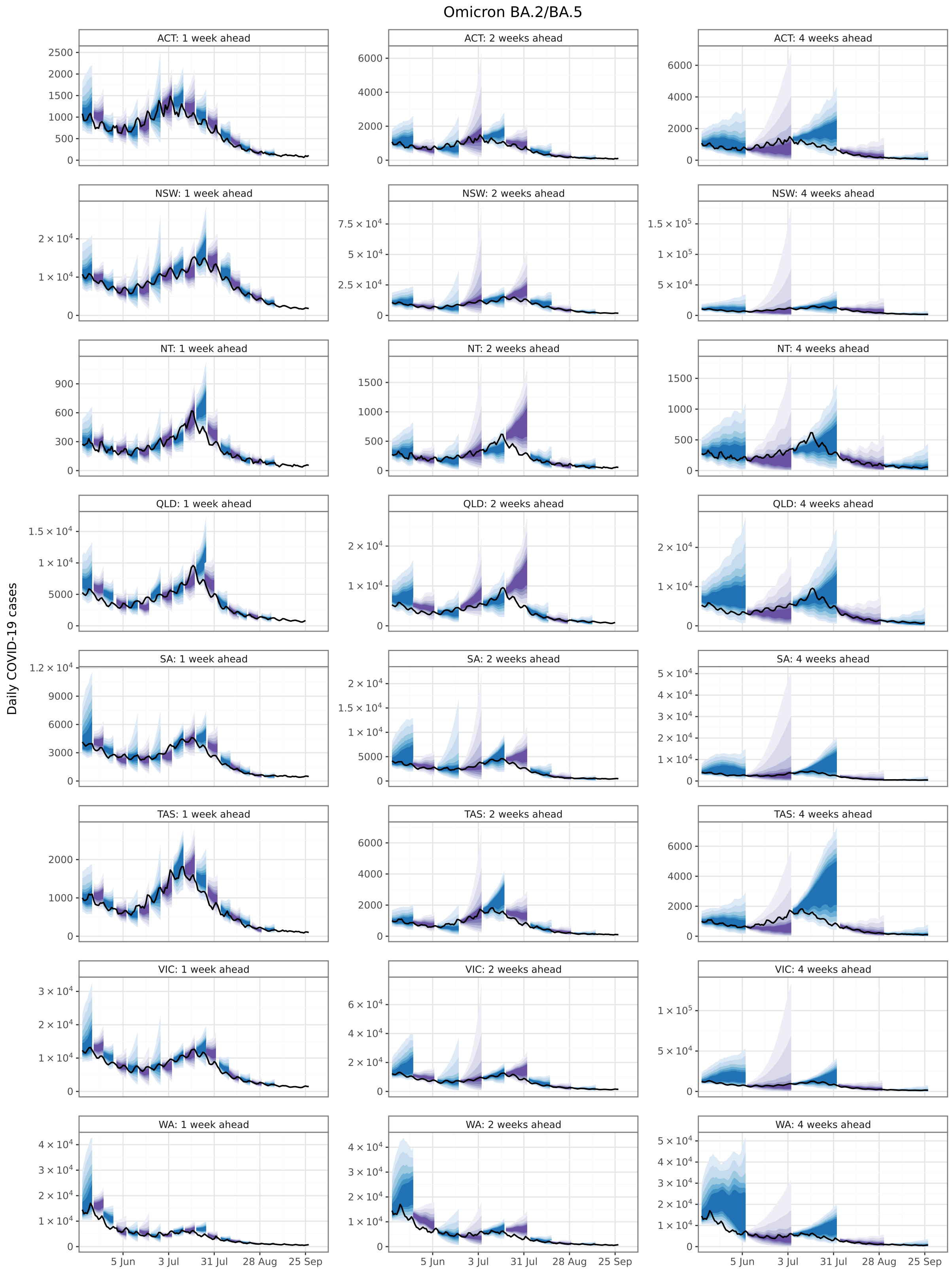

Figure D: Ensemble forecasts for the “Omicron BA.2/BA.5” period (2022).

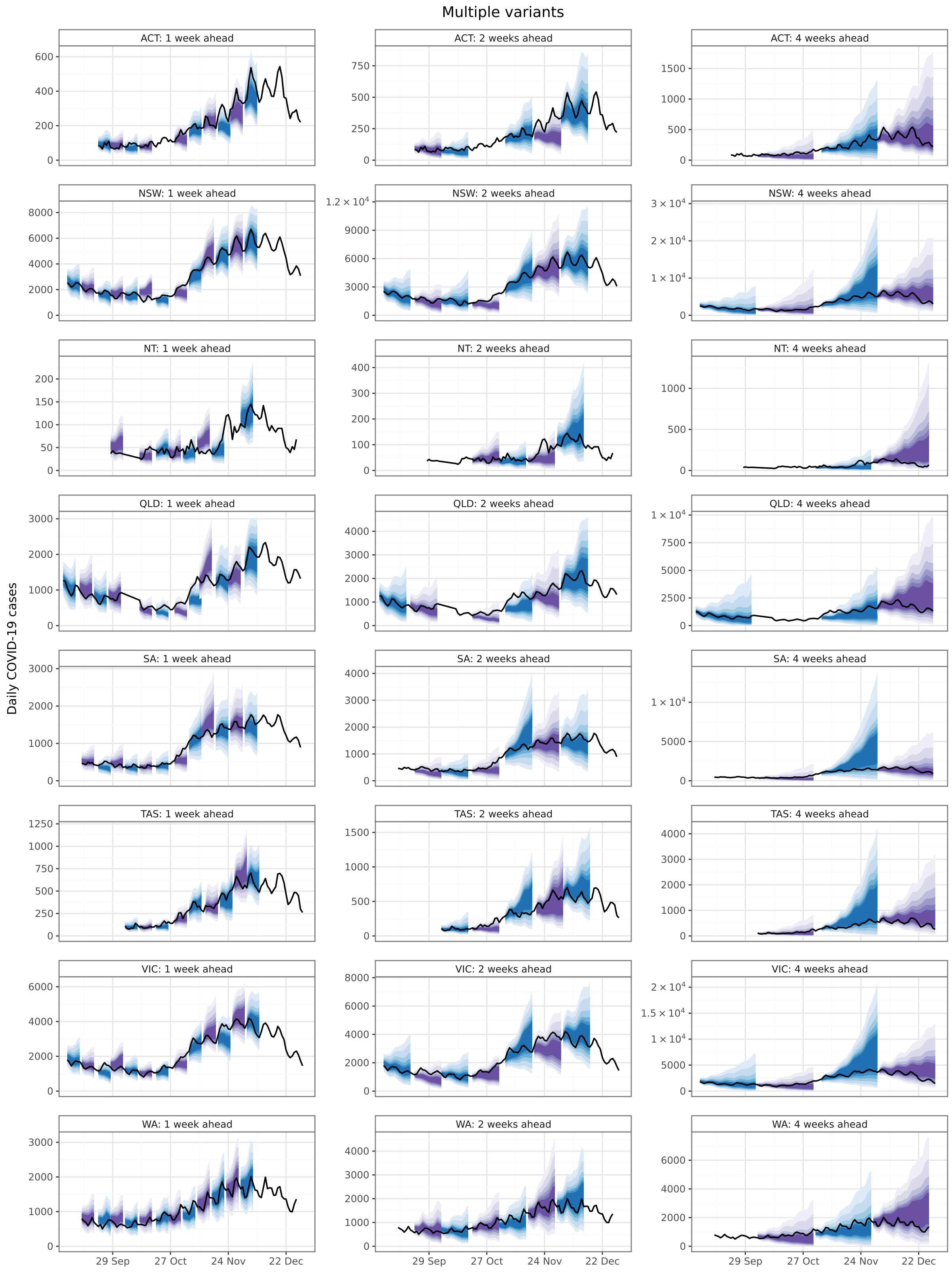

Figure E: Ensemble forecasts for the “Multiple variants” period (2022).

2 Forecast evaluations around observed epidemic peaks

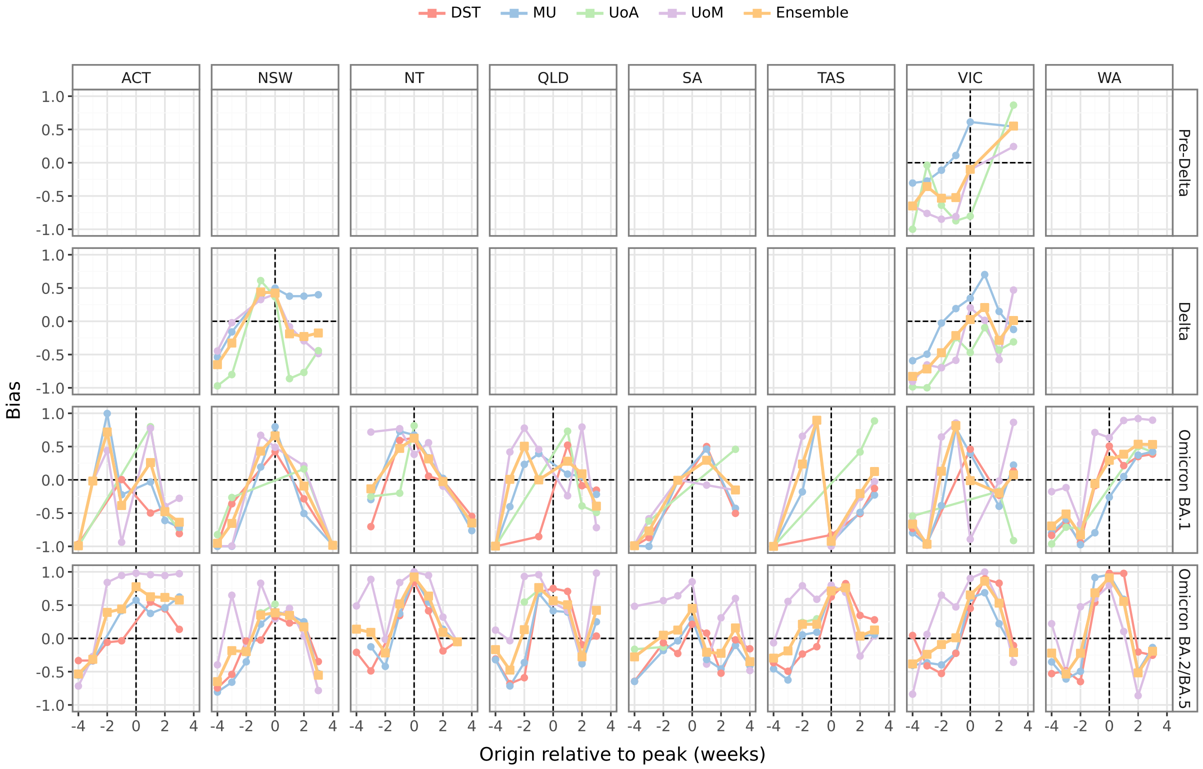

Figure F: Forecast bias for each model and the ensemble, shown for forecast origins within 4 weeks either side of the largest peak observed for each dominant strain (minimum 200 cases), with the exception of the "Multiple variants" period where activity was flatter and observed peaks occurred towards the very end of the study period.

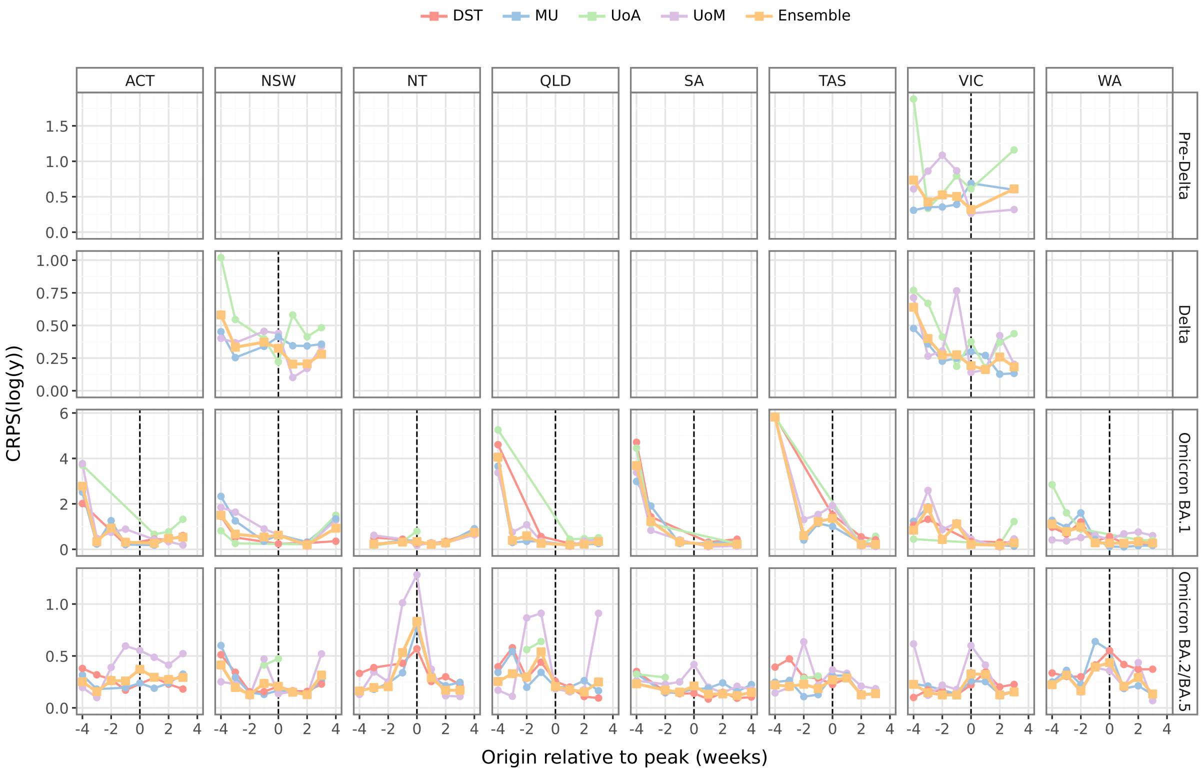

Figure G: Mean forecast CRPS for each model and the ensemble, shown for forecast origins within 4 weeks either side of the largest peak observed for each dominant strain (minimum 200 cases), with the exception of the “Multiple variants” period where activity was flatter and observed peaks occurred towards the very end of the study period.

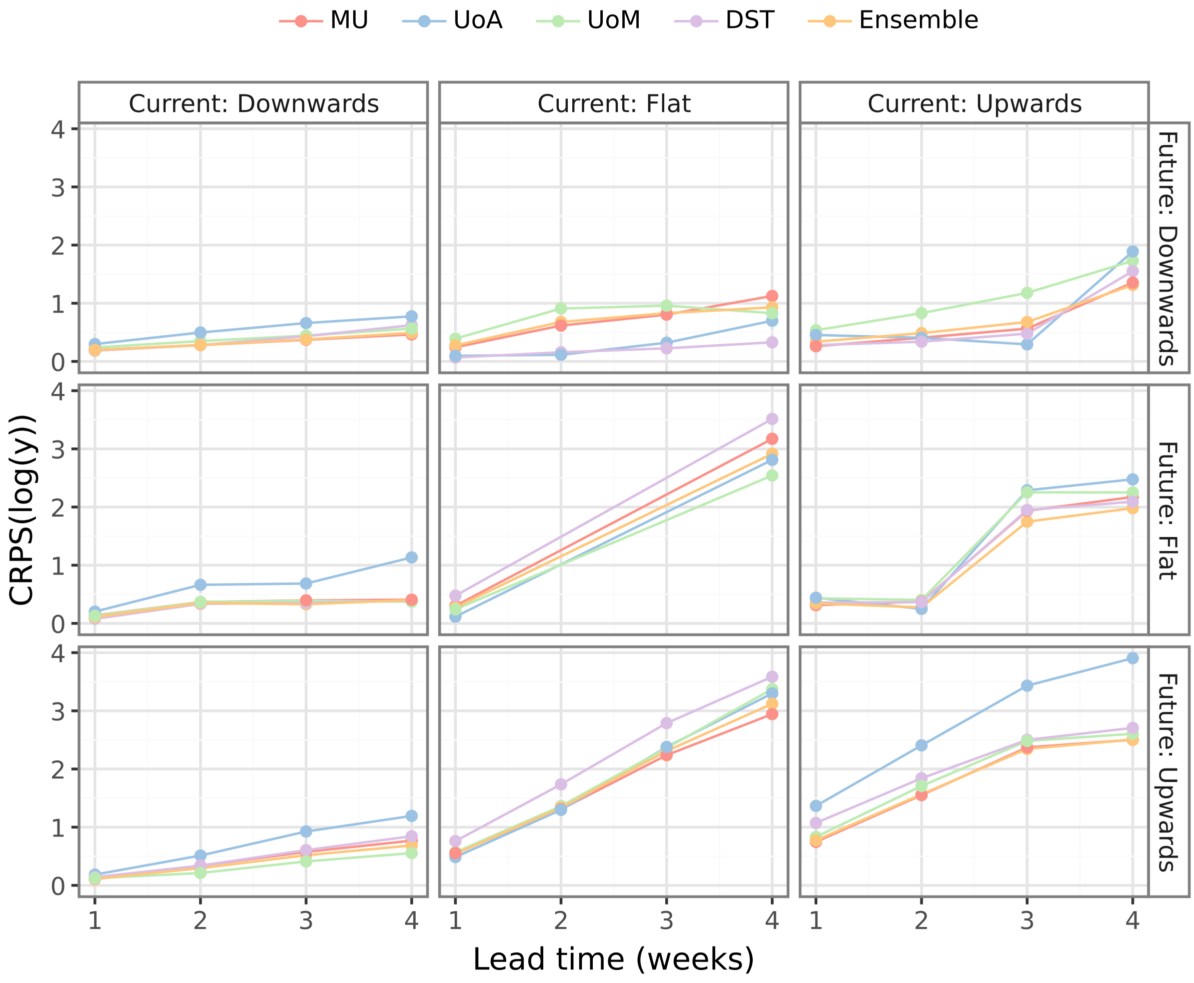

Figure H: Mean CRPS values for predictions made within 5 weeks before/after the peak observed in each jurisdiction for Omicron BA.1. Values are shown for each model, for each week of the forecast horizon, and categorised by the trend in the most recent case data (columns: downwards, flat, upwards) and the trend in the data over the corresponding week of the forecast horizon (rows: downwards, flat, upwards).

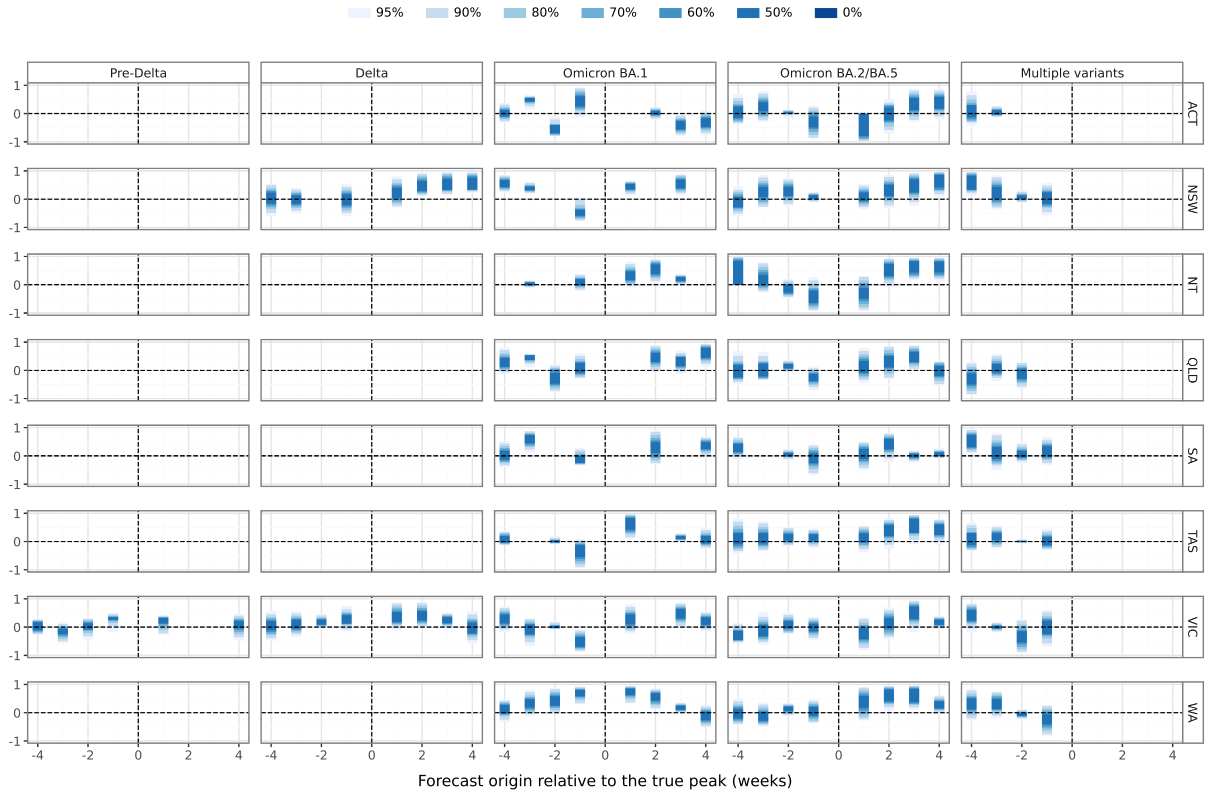

Figure I: The correlation between forecast trajectories and the ground truth case counts, calculated for forecasts where the true peak occurred within the four-week forecast horizon (negative x-axis values) or the four weeks prior to the forecast (positive x-axis values).

3 Model rankings for each ensemble forecast

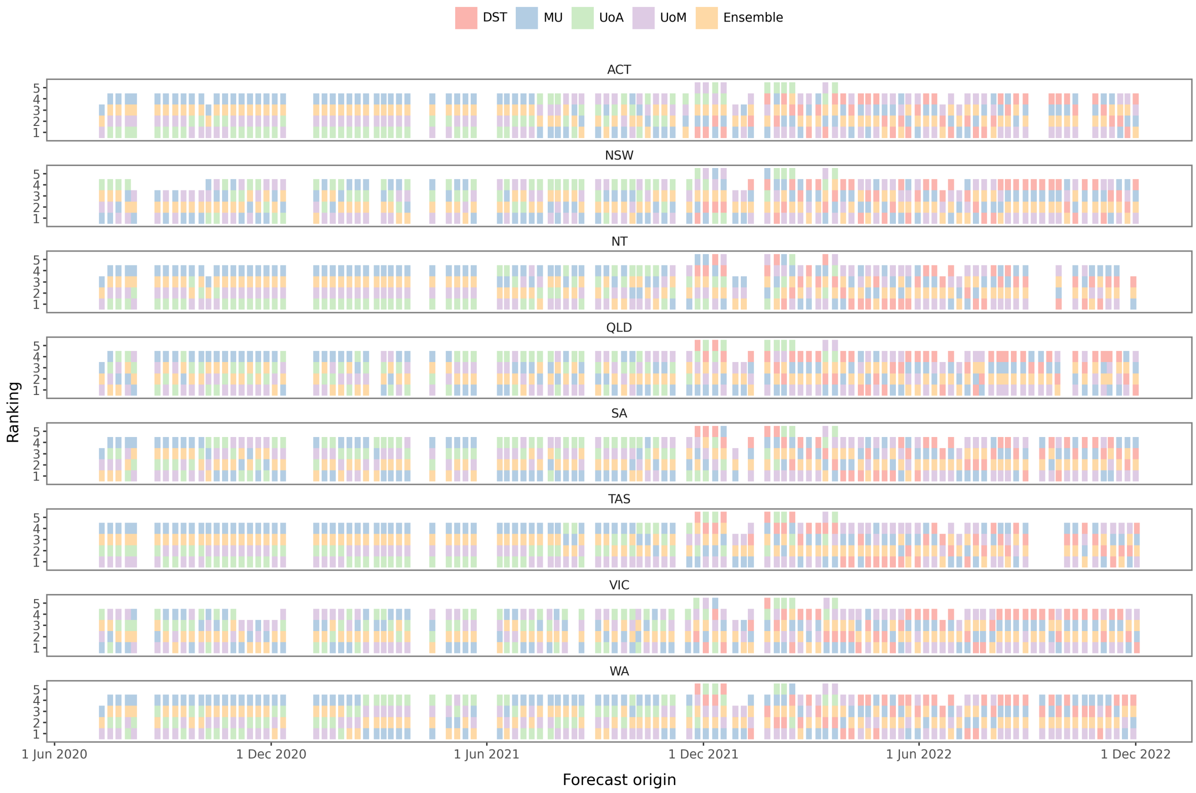

Figure J: The model forecast rankings for each ensemble forecast.

4 Model skill scores for the Pre-Delta wave in Victoria

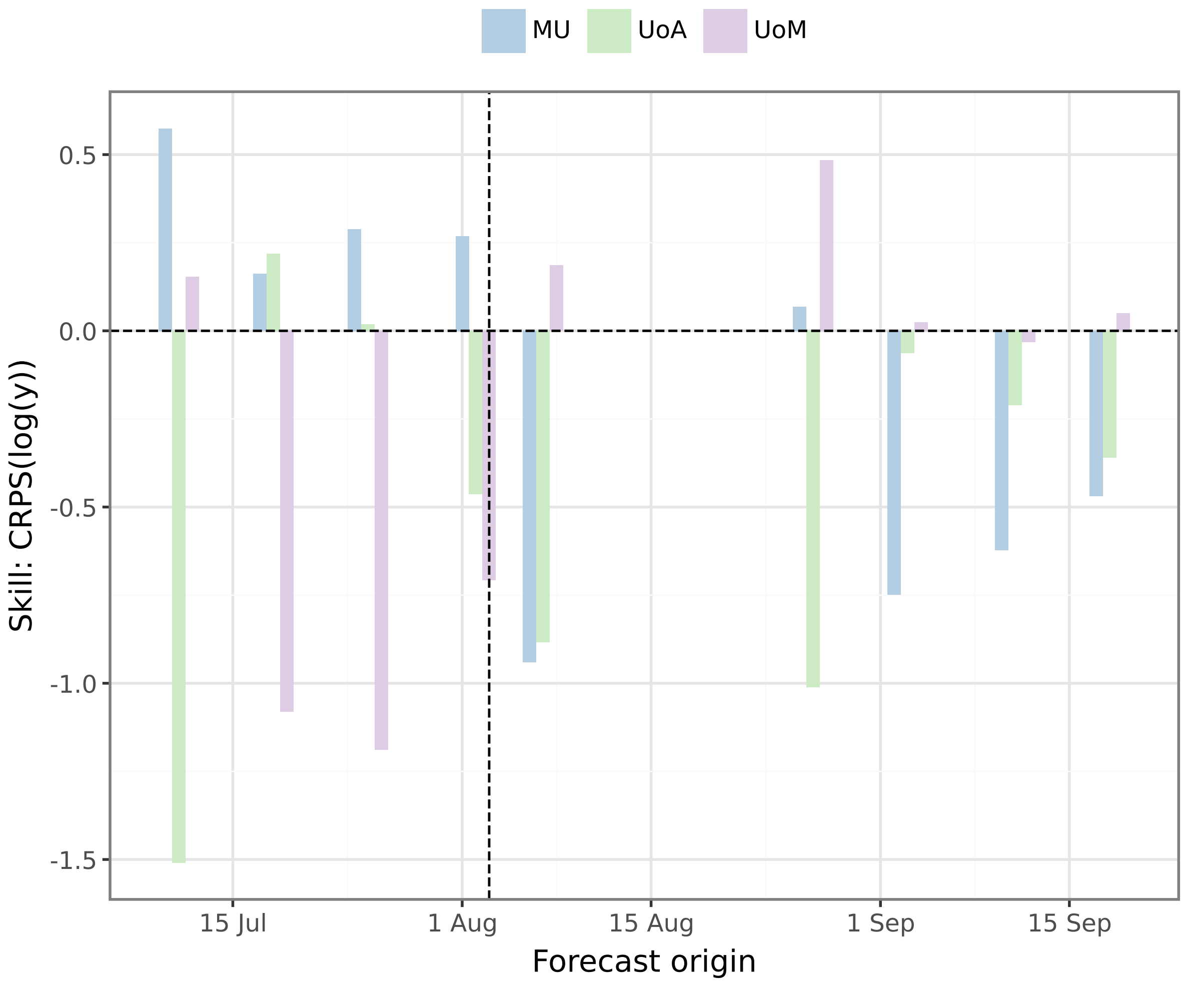

Figure K: Model skill scores during the second wave in Victoria (May to October 2020) relative to the ensemble forecast. The true peak occurred on 3 August 2020, as indicated by the vertical dashed line. Skill scores were calculated using CRPS on log-transformed values.

5 Probability integral transform (PIT) histograms

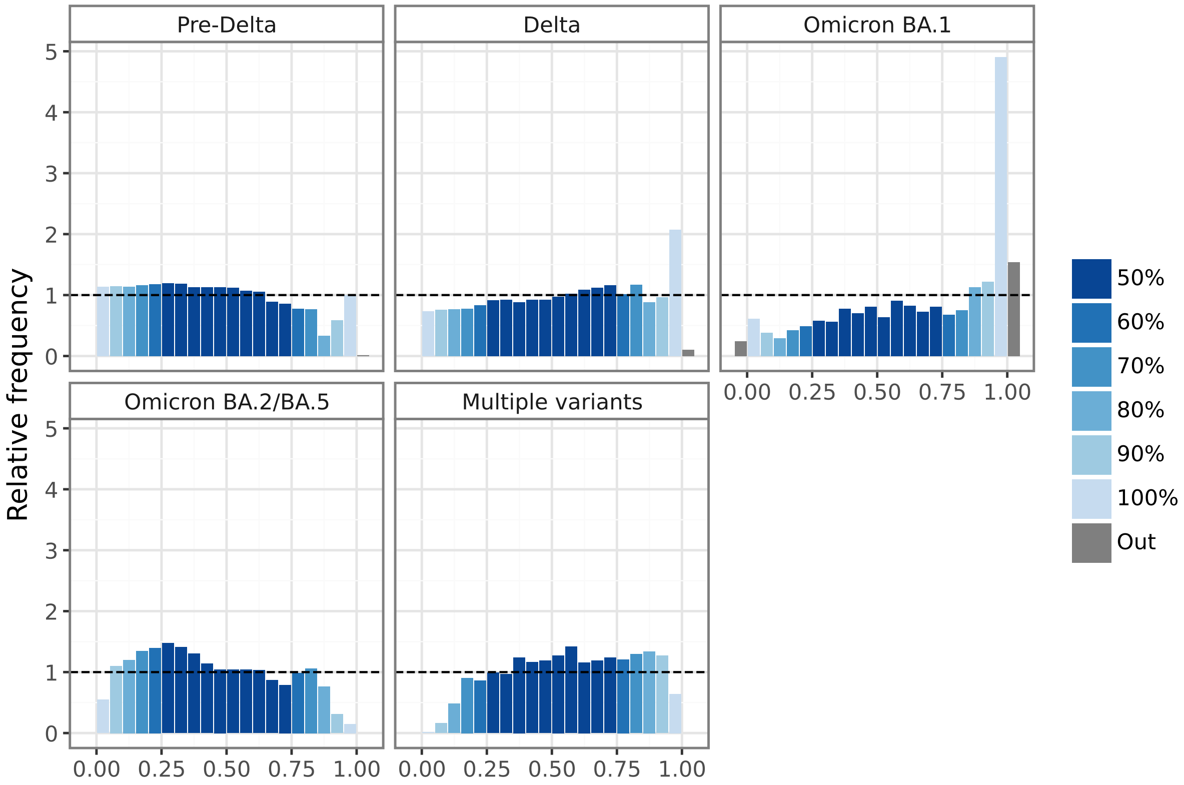

Figure L: Probability integral transform (PIT) histograms showing ensemble forecast coverage for each period where disease activity was dominated by a particular strain or group of strains.

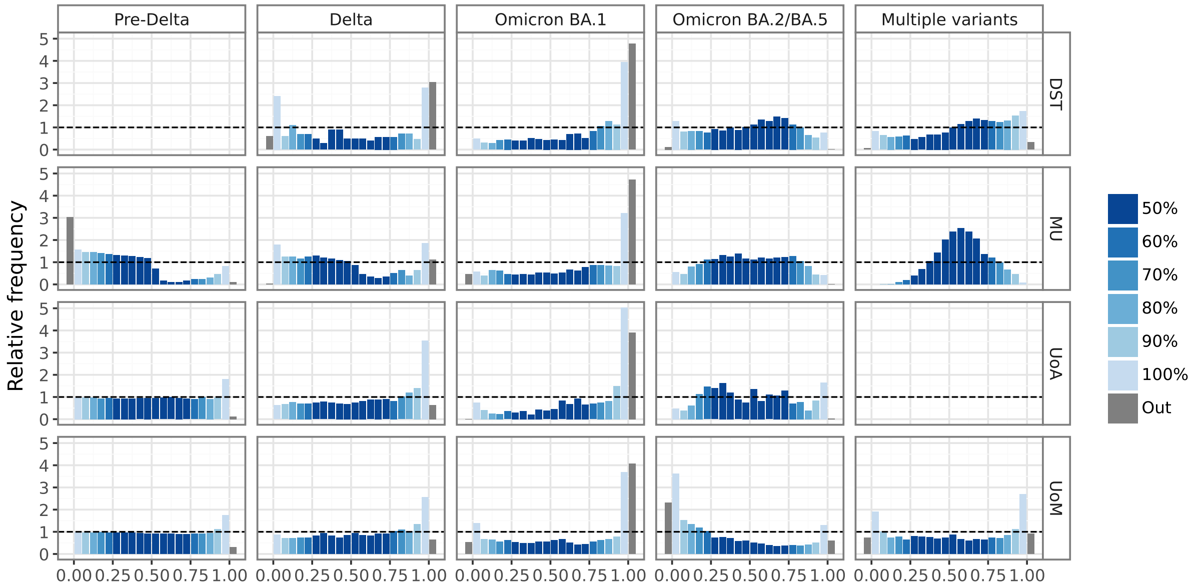

Figure M: Probability integral transform (PIT) histograms showing forecast coverage separately for each model in the ensemble, for each period where disease activity was dominated by a particular strain or group of strains.

6 Marginal quantile and CDF calibration plots

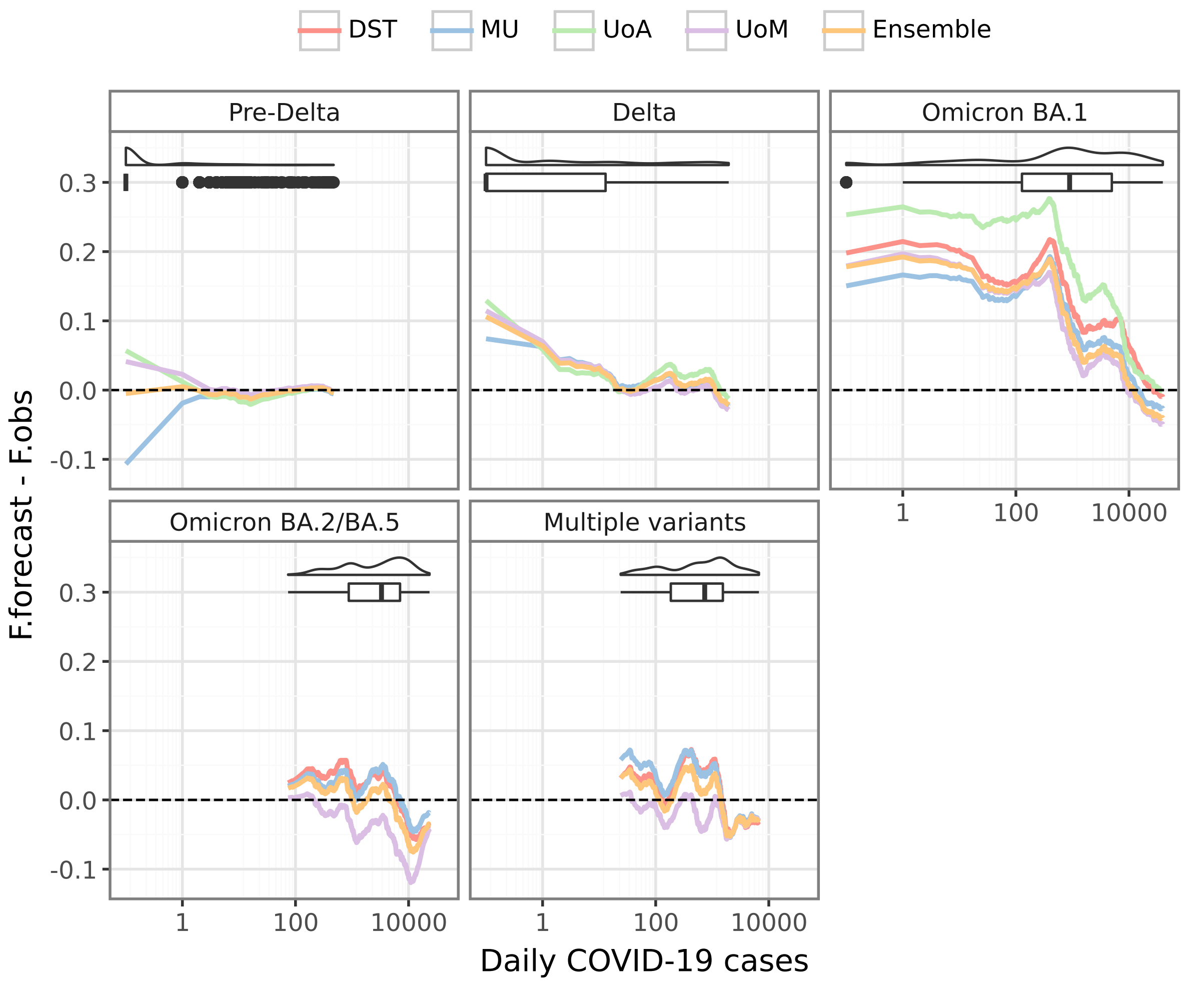

Figure N: The marginal CDF calibration for each model, shown separately for each period where disease activity was dominated by a particular strain or group of strains. All models over-predicted zeros during the Delta and Omicron BA.1 periods, and under-predicted the highest case counts during the Omicron BA.1 period.

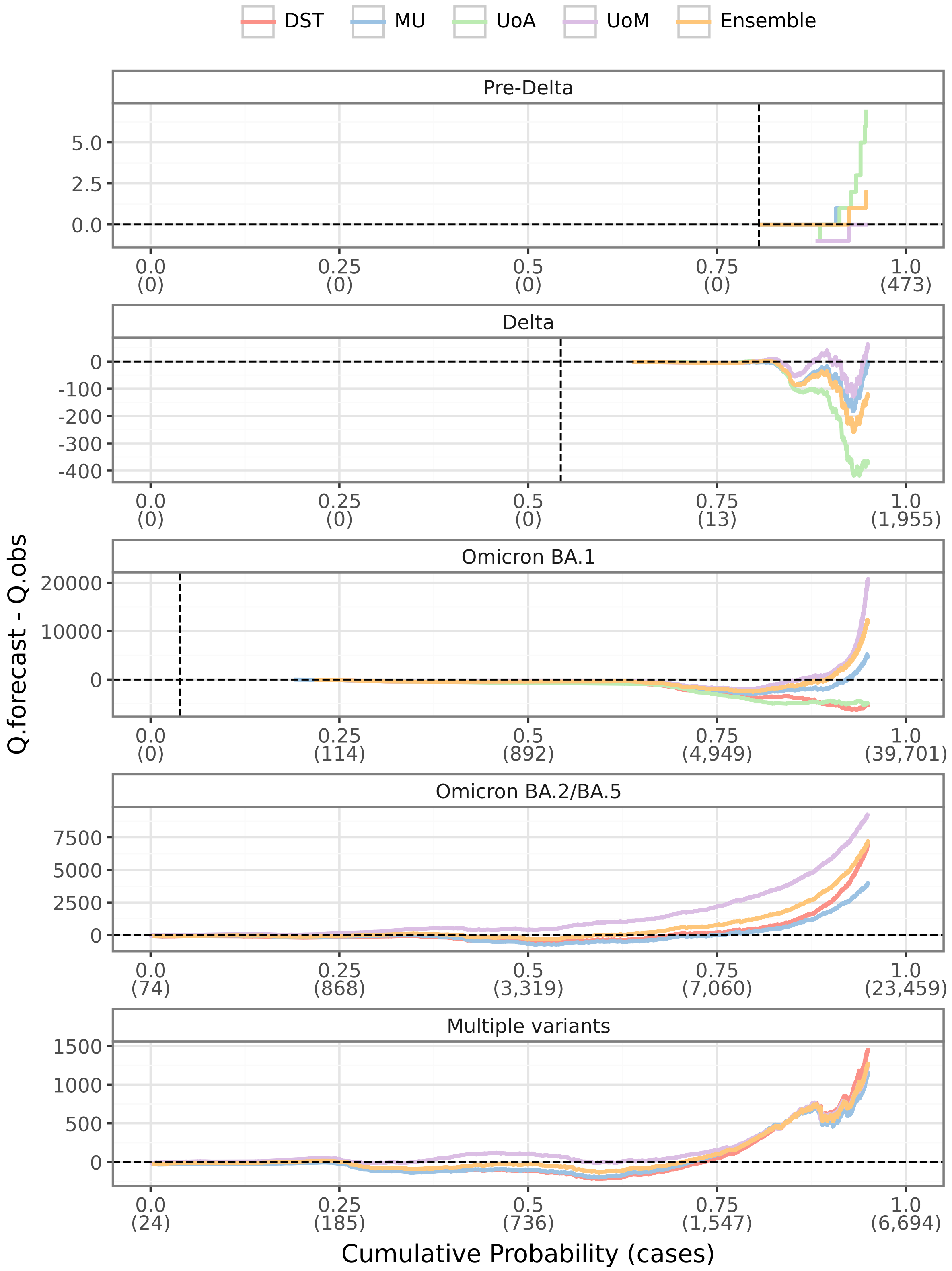

Figure O: The marginal quantile calibration for each model, shown separately for each period where disease activity was dominated by a particular strain or group of strains.

7 CRPS values for each dominant strain

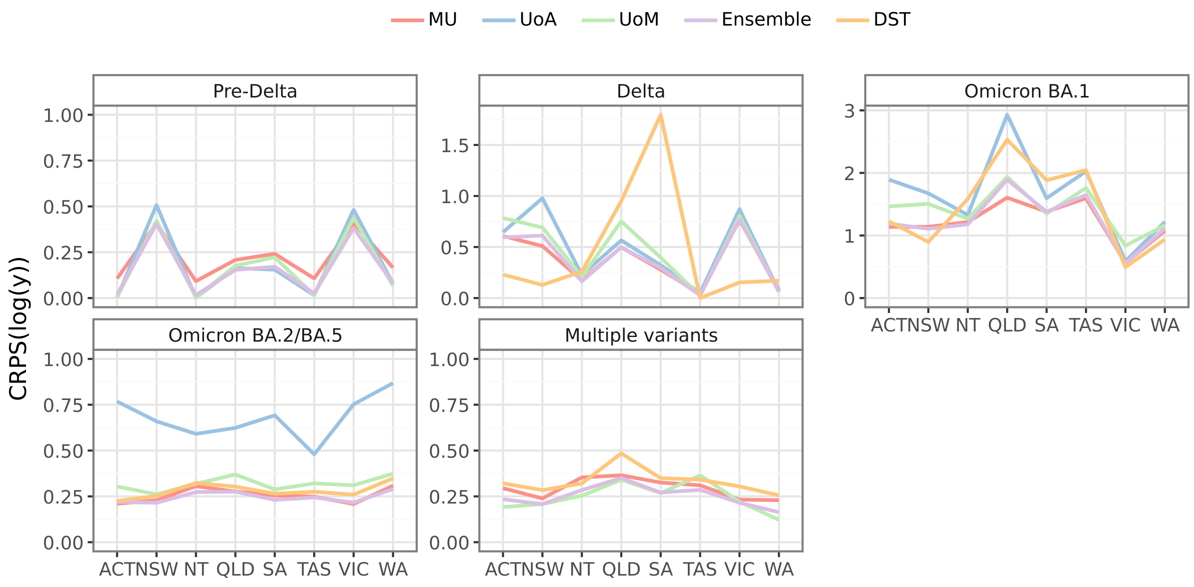

Figure P: Mean CRPS for each model, and the ensemble, shown separately for each jurisdiction and for each dominant strain.
