# Supplementary material for: Ensemble forecasts of COVID-19 activity to support Australia’s pandemic response: 2020–22
Source: PLoS Comput Biol. 2026 Apr 22;22(4):e1014199. doi: 10.1371/journal.pcbi.1014199 (PMC13124057; doi:10.1371/journal.pcbi.1014199)
Supplement: Attachment — Submitted filename: response-letter.pdf [file pcbi.1014199.s003.pdf]

We thank the editor and reviewers for their careful consideration and detailed comments on our manuscript, and for expressing their enthusiasm regarding our work and the role it played in Australia's COVID-19 response. Please find our responses to these comments listed below, highlighted by red brackets as per this paragraph.

Reviewer #1

This manuscript presents an overview and evaluation of the ensemble forecasting effort that supported Australia's COVID-19 response between 2020 and 2022. Several aspects of the presentation and methodological exposition require improvement before publication.

More formal presentation

Much of the description of the ensemble construction and evaluation is expressed verbally rather than formally. The reader is left with only a broad qualitative sense of how the ensemble was produced rather than a rigorous definition that would allow replication or scrutiny. This reviewer strongly recommends formalizing the ensemble description with clear notation and explicit equations. For example, define the ensemble forecast X as a sample over contributing model distributions ϕ_i, i.e.:

$$p = \sum_i w_i \phi_i(t),$$

with the sampling procedure clearly explained. As is, statements such as "weights were allocated to each team rather than each model iteration" are extremely vague. It should be clear what the random variable represents, over what the averaging occurs, and how dependencies among trajectories are handled. Without formalization, it is difficult to assess the ensemble's statistical coherence. This reviewer would understand an the informal presentation were the article aimed at a policy-oriented audience, but this is a computational journal – formalism is not only acceptable, it is expected.

As suggested by this reviewer, we have provided a mathematical definition for the ensemble that demonstrates how the forecasts contributed by each team, and the separate model iterations, were combined.

We have also provided much more detail for our measures of absolute forecast performance (CRPS, §2.5), relative forecast performance (CRPS skill scores §2.5), forecast calibration (credible interval coverage, §2.6), and forecast bias (§2.7). As noted by this reviewer and other reviewers, our original methods section did not provide sufficient details of these measures, and we hope that the revised methods section has addressed their concerns.

Evaluation metrics

The notion of "coverage" of credible intervals is used repeatedly but without a formal definition of what constitutes a credible interval in this context. This reviewer suggests clearly

supplementing the probabilistic measures (CRPS, bias, coverage) with more straightforward metrics, like absolute or relative case-count discrepancy, mean absolute error, or median absolute deviation. Such data should be presented both graphically and numerically.

In our revised methods section (§2.5) we now explain that CRPS on the natural scale is a generalisation of mean absolute error to probabilistic forecasts, and that CRPS on the logarithmic scale is a generalisation of relative error to probabilistic forecasts. We have also added definitions for credible intervals and credible interval coverage (§2.6)

The metrics that the reviewer has suggested are appropriate measures for point forecasts, and can be applied to, e.g., the mean or median value of a probabilistic forecast. However, these metrics ignore the uncertainty in the forecasts, and this uncertainty is a critical feature of the forecasts and their potential value to public health stakeholders. To encourage probabilistic decision-making, forecast intervals (deciles) without point forecasts were reported to stakeholders and so point-based evaluation metrics are not appropriate for evaluating the forecasts that were produced. Probabilistic forecasting is the standard in many other areas of science, including economics and weather forecasting, and the inherent difficulty in forecasting epidemics makes it even more important to properly reflect the uncertainty in evaluating forecast accuracy (Held et al., Statistics in Medicine, 2017).

Model identity and comparability over time

The manuscript explicitly notes that individual modeling teams (UoA, UoM, DST, etc) updated or substantially revised their models during the study period, and that inclusion decisions were based on weekly peer review. Given these changes, it seems questionable to treat "UoA" or "UoM" as single, stable models during the ranking analyses (Fig. 6-7).

If a team's model underwent structural revisions that materially altered its behavior, those forecasts effectively derive from different models. Treating them as one entity may confound the interpretation of the ranking and bias analyses. The authors should clarify whether their results aggregate across fundamentally distinct model versions and, if so, discuss the implications. It may be more defensible to define "model iteration" as the unit of analysis.

This would be a complex undertaking, as there were ongoing tweaks and modifications to the models, and there isn't necessarily a clean divide between one model iteration and the next. In the most extreme case, we would have to evaluate one or more model iterations from each team for each of the five dominating strain periods, and this would make summary metrics and conclusions difficult to present and interpret.

We have added the following text to the method section (§2.4, Ensemble forecast construction):

> When a team provided forecasts from multiple model iterations, we treated these forecasts as arising from a single model with a discrete

hyper-parameter (i.e., having a different value for each model iteration), and averaged over the iterations when including the team's forecasts in the ensemble. We believe that this was the most sensible approach to take, particularly when our primary interests were to evaluate how well each model performed relative to the other models, and relative to the ensemble. The modifications made to each model throughout the study period were much smaller than the methodological and structural differences between the models. Accordingly, the addition or removal of one model iteration impacted the relative weights of all model iterations from that team, but did not affect the weights allocated to other teams.

We note that COVID-19 ensemble forecasts in the United States of America and the European Union had open submission policies; they accepted forecasts from large numbers of teams, and only required a brief description of each model (typically 1–2 sentences, see Table S1, Cramer et al., PNAS 2022), with no requirement that teams provide notice if they made any changes to their models. That being said, a number of teams that participated in COVID-19 ensemble forecasts in the United States of America did notify the organisers that they had updated their models during the study period (Figure S1, Cramer et al., PNAS 2022; also see the footnotes to Table S1). Updates included switching between data sources, to changing how models were fit to past data, to incorporating mobility in models, to using hierarchical approaches that shared information between regions, and changing how initial conditions were estimated. In all cases, the performance measures for each team were calculated across the entire study period.

The European COVID-19 ensemble forecast analyses did not report whether any of the participating teams made changes to their models during the study period (Sherratt et al., eLife 2023).

Additional concerns and recommendations

1. The discussion of peer review and human judgment in excluding "suspect" models is interesting but could benefit from a more rigorous treatment of how this subjective step affects ensemble uncertainty. Is there a quantifiable impact of exclusion decisions on forecast variance or calibration?

As we describe in the methods section (§2.3, Inclusion/exclusion criteria):

> Models were excluded when their outputs differed dramatically from the consortium's collective expectations and the current epidemiological assessment, or when a technical issue was identified by the modelling team or by the peer review process.

We included all of the contributed forecasts by default, and only excluded those that were considered extremely implausible or when a technical issue was identified. As we describe in the result section (§3.1) the most common reason for exclusion was for a model to exhibit large and systematic biases

in forecasts produced in previous weeks. In these cases, excluding the model from the ensemble consistently reduced the variance of the ensemble forecast, because the excluded model produced forecasts that had little or no overlap with the other models.

Since the ensemble forecast coverage was generally very good (with the exception of the Omicron BA.1 wave), it is likely that adding these excluded models into the ensemble would have adversely impacted the forecast coverage. In the specific case of the Omicron BA.1 wave, no model produced well-calibrated forecasts (as shown in Figure 4). Some models did not contribute to the ensemble forecasts from late December 2021 until early January 2022, but this was due to staff taking leave over the Christmas holiday period, rather than teams submitting forecasts that we decided to exclude on any grounds. We have added this observation to the results section (§3.1):

> Due to staff leave, some teams did not provide forecasts over the Christmas 2021 holiday period (during "Omicron BA.1").

We do highlight one specific instance in the results section (§3.2, end of first paragraph) of including a model which had a noticeable effect on the ensemble forecast:

> The extremely broad intervals for the Victorian forecast for 15 March 2022 arise from a single model, which was assessed as highly uncertain but consistent with the data, and included in the ensemble that week.

Please note that we are providing a record of the work as it was conducted and used to inform policy in real-time. It would be a substantial undertaking to extract the rationale for each exclusion decision from the weekly situational assessment reports submitted to the Australian Goverment and compare the coverage of each hypothetical ensemble forecast against that of the ensemble forecasts that were reported to government.

2. The discussion attributes reduced accuracy during Omicron BA.1 largely to epidemiological volatility and data limitations. While plausible, some quantitative decomposition (e.g., sensitivity to case ascertainment assumptions) would make this more convincing.

This wasn't part of the study itself, but two of the three teams participating at that time were using mechanistic models, and they each undertook retrospective analyses (unpublished) to see if they could obtain improved forecasts of the Omicron BA.1 peak. We now mention this in the discussion section (§4.3, Study limitations):

> Two of the three teams that participated during the "Omicron BA.1" period (DST and UoM) used mechanistic models that explicitly incorporated case ascertainment and immunity dynamics.

In separate retrospective analyses conducted after this period (unpublished), each team explored adjustments to case ascertainment and immunity assumptions, on the grounds that this might result in smaller and earlier predicted peaks, closer to the observed peaks. However, both teams reported when that these adjusted models were fit to the reported (pre-peak) case data, estimates for other parameters also changed in response to these adjustments, and this resulted in similar forecasts to the original models (i.e., peaks were larger and later than observed).

3. The manuscript occasionally uses "forecast performance," "bias," and "skill" interchangeably. These should be defined explicitly, as readers from outside infectious disease modeling may not share the same intuitive understanding.

   This is a fair point, and consistent with the reviewer's earlier comments about formal presentation of methods and evaluation metrics.

   We have revised the methods section (see our response to this reviewer's first comment). We now describe our use of CRPS values as *absolute* measures of predictive performance, of CRPS skill scores as *relative* measures of predictive performance (with respect to the ensemble), of credible interval coverage as a measure of forecast calibration, and bias as a measure of consistent over/under-prediction.

This reviewer would like to commend the authors for this work, and looks forward to reading an improved version in the near future.

We thank the reviewer for their careful consideration of this manuscript and their enthusiasm for our work.

Reviewer #2

Firstly, I would like to thank the authors for their service and work throughout the pandemic. Secondly, I would like to thank them for the submission that reflects on their results and the impact on public policy in Australia – I thoroughly enjoyed reading the manuscript and found it refreshing that they are willing to reflect on their work throughout the pandemic. This manuscript focusses on the results and reflects of an ensemble method using for forecasting daily COVID-19 cases in Australia, combining models from 4 different modelling groups who were asked to contribute towards pandemic efforts, and describes the consortium's internal process for the exposition of the results for a wider audience and its successes. In particular, the manuscript demonstrated some results of the ensemble model and showed some specific case studies where the approach performed well (and often better than the individual models), then went into a discussion on the findings, strengths and limitations, and the implications. Whilst I enjoyed reading the manuscript and its reflections, it does not seem to be presenting any novel mathematics or even a novel application of the mathematics, it seems to be only novel as a geographic location. It is a good account of the history of the consortium and the modelling efforts of the time, but that seems to be it? Whilst that, in itself, is not enough reason to reject the manuscript (there are plenty of examples in PLoS Comp Biol that are doing this), I think the manuscript needs a bit more care - the manuscript reads like a large situational report, rather than an academic journal article.

We thank the reviewer for their interest in, and appreciation of, this work. In response to their comments, and those of the other reviewers, we have made a number of revisions to this manuscript that we believe should resolve this reviewer's concerns about this reading like a report.

In particular, in the introduction we now explicitly state the novel aspects of this work (with reference to the USA and European COVID-19 forecasting hubs). We have expanded the methods section, which now includes a mathematical definition for the ensemble and with much more detail about our measures of absolute forecast performance (CRPS), relative forecast performance (CRPS skill scores), forecast calibration (credible interval coverage), and forecast bias. We also highlight that clear and effective communication of forecast outputs to stakeholders was of paramount concern, since the purpose of this work was to directly support Australia's COVID-19 response, and our reflections on the reporting and visualisation of these forecasts are an important aspect of this study.

Consistent with articles published in PLOS Computational Biology and other journals (as noted by the reviewer) we are providing a record of the work as it was used to inform policy in real-time. Further, all of the retrospective analyses presented here (e.g., of absolute and relative performance, calibration, bias, or rankings) are new results that were produced after the contract work was completed and provide valuable insights for future forecasting efforts.

My recommendation for this submission is Major Revision.
The major issues I have are as follows:

1. The introduction needs to have a more substantial literature review of ensemble models, where they have been used and to what effect.

We have added a paragraph that introduces ensemble forecasting (with reference to numerical weather prediction and climate forecasting), highlights the "forecast combination puzzle" that a simple average of candidate forecasts typically out-performed more complicated methods, and identifies the US CDC's FluSight competition (begun in 2013) as the major source of ensemble forecasting for infectious diseases prior to the emergence of COVID-19. We conclude this paragraph by observing that both the US and Europe established COVID-19 forecasting hubs, and that studies published by both hubs made similar findings regarding the robust performance of simple average ensembles.

2. The introduction needs to highlight explicitly what is novel about this work, and clearly highlight what the take home message is about the approach conducted.

We have revised the introduction to highlight the novel aspects of this work, as suggested by the reviewer:

> Here we present an analysis of the ensemble forecasts that were produced by the authors, under contract with, and reported to, the Australian Government. We demonstrate how the ensemble consistently out-performed the individual models that were included in the ensemble, and how these forecasts supported public health responses over 2020–2022. Because we were directly funded by the Australian Government to support Australia's public health responses, clear and effective communication of forecast outputs to stakeholders was of paramount concern, and we reflect on how we refined our reporting and visualisation over the study period. Relative to the USA and European COVID-19 hubs, distinguishing features of this work include our internal peer-review process, our detailed knowledge concerning the methods and implementation of each contributing model (due in part to our much smaller scale), and our use of daily case counts which, when compared to using weekly counts, adds complexity regarding test-seeking and diagnosis timelines. We conclude by highlighting the relative strengths and weaknesses of our ensemble approach, relative to the USA and European COVID-19 hubs.

3. The method section should outline more information about how the ensemble model is actually constructed (there are random bits of information throughout the manuscript, such as equal weighting in Section 4.2).

We agree, and have revised the methods section to explain this in detail (as we outline in our earlier response to a similar suggestion from Reviewer #1, "More formal presentation").

4. There seems to be something interesting that the authors make a large point about: "the models were evaluated against the most recent data rather than competing

against the other models . . . , with the purpose of deciding whether the model outputs were likely to be a reliable basis for decision making." It is not clear from the text why this is relevant and important?

This is a philosophical point, which is nicely explained by Gelman and Shalizi (reference 33, British Journal of Mathematical and Statistical Psychology, 2012). In brief, we do not believe that any of the participating models truly captures the real-world system, so the models are not competing to be the "true" model, and we are not using performance metrics to undertake model selection.

We have added the following text to the methods section (§2.3, Inclusion/exclusion criteria):

> In other words, because we did not believe that any participating model truly captured the real-world system, we therefore did not use performance metrics to undertake model selection. Rather, teams were able to use these metrics to inform model development.

5. There should be a discussion about when one should produce an ensemble model and when one should not.

This is a good point. For our study, at each week we decided that forecasts from multiple teams were plausible and were selected for inclusion in the ensemble. Under these circumstances, we would expect an ensemble forecast to out-perform any of the individual forecasts, and so we always produced an ensemble forecast.

We have added the following details to the methods section (§2.4, Ensemble forecast construction) about the expected value of an ensemble forecast and our concerns about relying too much on any single model:

> The use of equal weights for each team was strongly motivated by the risk of a sub-optimal forecast supporting a poor decision/recommendation. An ensemble consistently performs better than a single model, and there is a large body of evidence in the forecasting literature that it is very hard to out-perform an equal-weight ("simple average") ensemble; this is known as the "forecast combination puzzle" [21,22]. This reliability of a "simple average" ensemble was more valuable for our purposes than any potential (and likely very small) gain in performance from using a more complex weighting scheme.

We also added the following text to the results section (§3.1, Models included in each ensemble forecast) to highlight that we always accepted forecasts from multiple teams:

> At each week of the study period, we accepted forecasts from multiple teams (with a minimum of two teams) and always produced

an ensemble forecast, since an ensemble is expected to out-perform any individual model.

6. Section 4.4: a lot of this implies that having a small ensemble is better, which I don't think is necessarily true in general and could be quite misleading for readers. It would be better if the authors also included the positives that came out of the other two hubs, so the reader can make an informed decision themselves.

We did not intend to imply that a smaller ensemble is better than a larger ensemble, but rather to point out that there are diminishing returns with the inclusion of greater numbers of models in an ensemble, and that the smaller scale of our ensemble had some positive benefits.

We have added a paragraph to Section 4.4 that discusses the benefits of a greater number of models, and highlight that our average of 3.03 models is smaller than the minimum of 4 recommended by Fox et al., EID, 2024. We also point out in the penultimate paragraph that the advantages of our peer review process, while particularly valuable given the small number of teams participating in our ensemble, may not confer much benefit when the number of teams is much larger, and may not be feasible at times of high stress and urgent delivery schedules.

The minor issues I have are as follows:

1. How and why did the authors impute missing symptom onset dates? Imputing in general is widely criticised (e.g., Dettori et al. and Jørgensen et al.).

These papers refer to cohort studies, which is a very different context. For example, such studies typically aim to estimate the effect of an exposure (e.g., treatment) on an outcome, subject to individual-level factors (e.g., age and sex). When there are missing data, analyses can be restricted to only those patients from whom complete data are available, or the missing data can be imputed and this may impact the analysis results (as discussed by the papers provided by the reviewer).

In contrast, we used de-identified line lists of reported COVID-19 cases in Australia to estimate the underlying infection dynamics and predict the future trends in these data. We aggregated COVID-19 cases by symptom onset date rather than notification date, because this provides more information about recent transmission events and the current growth rate (Gostic et al., PLOS Comp Biol, 2020). If we didn't impute missing symptom onset dates, we would have had to use less of the cases/infections data, and would still be making the same assumptions about the relationship between the date of infection and the date of symptom onset. Similar inference methods are included in state of the art R packages, such as `EpiEstim` and `EpiNow2`.

The imputation method is described in reference #19, which we cite at the

end of the relevant sentence in the methods section (§2.1) before summarising how often symptom onset dates were reported:

> We imputed missing symptom onset dates and estimated reporting delays using a time-varying delay distribution to characterise the duration between symptom onset and case notification [19]. Symptom onset dates were reported for 82% of cases in 2020. As Australia's response pivoted to "reopening" the country, and daily case counts greatly increased, symptom onset reporting decreased to 48% of cases in 2021, and 38% in 2022.

This imputation method has been previously described by Golding et al., (ref [19]) who developed a semi-mechanistic modelling framework to estimate the effective reproduction number of COVID-19 from Australian case data. Here is a brief description of the method, taken from ref [19]:

> "For a given date and state/territory, the empirical distribution of delays from symptom onset to notification is computed from cases with symptom onset falling within a time window around that date, with the window selected to be the smallest that will yield at least 500 observations; but constrained to between one and eight weeks.
>
> Where a state/territory does not have sufficient cases to reliably estimate this distribution in an eight week period, a national estimate is used instead. Specifically, if fewer than 100 cases, the national estimate is used, if more than 500 the state estimate is used, and if between 100 and 500 the distribution is a weighted average of state and national estimates.
>
> The national estimate is obtained via the same method but with no upper limit on the window size and excluding data from Victoria since 14 June [2020], since the situation during the Victorian outbreak after this time is not likely to be representative of surveillance in states with few cases."

2. Figure 1: it might be good to highlight more substantial changes in the pandemic curve here (e.g., vaccination). Can the authors also clarify that it is correct that there were less than 10 cases from late December 2020 to approximately June 2020? If that is true, I would suggest writing something in the text as this was not expected (and I suspect would not be expected by readers outside of Australia).

We did experiment with more heavily annotated versions of Figure 1, but there were too many relevant details for it to remain legible (in particular, there were several stages of vaccine roll-out and related changes such as removal of quarantine requirements). For this reason, we have instead provided a separate timeline of these events in Table 1.

And yes, we can confirm that the national case counts shown in Figure 1 are correct — over the period where Australia's COVID-19 response focused on preventing local transmission, local elimination was achieved for extended periods of time. We state this in the introduction:

> Australia's initial COVID-19 response focused on preventing local transmission and, despite a steady influx of imported cases, local elimination was achieved for prolonged periods throughout 2020 and 2021, punctuated by several waves of local transmission. Over the 8 months from 1st October 2020 to 1st June 2021 there was a total of 874 reported cases, of which 345 were in the state of Victoria.

And we also highlight in the results section (§3.5, "Delta: June to December 2021") that:

> The onset of the Delta variant in June 2021 marked the first instance of sustained local COVID-19 transmission for most Australian jurisdictions, and public health responses focused on preventing local transmission.

3. In the formula for forecast bias, what are Y_t and y_t? The manuscript refers to bias quite a bit so it would be appropriate to include more information about it. Similarly for CRPS, the authors should include more details.

We agree that methodological details were lacking. As outlined in our response to Reviewer #1 ("More formal presentation"), we have added these details to the methods section and hope that this reviewer finds the revised content to be sufficiently thorough and complete.

4. Figure 3: why do the credible regions look so disjoint? I think it is due to the refreshing of model outputs? If so, please make this clearer. I would also suggest making the y-axis the same for either all the figures or for the ones the authors want the reader to compare (probably the same y-axis for each of the regions).

As suggested by the reviewer, we now use a consistent y-axis scale for each jurisdiction.

This figure shows ensemble forecasts over a 3-month period in four jurisdictions (rows). For each jurisdiction, the left-most panel shows 1-week forecasts (i.e., the predictions 1–7 days into the future for each forecast), the middle panel shows 2-week forecasts (i.e., the predictions 1–14 days into the future for every second forecast), and the right panel shows complete 4-week forecasts (i.e., the predictions 1–28 days into the future for every fourth forecast). For clarity, we have added these forecast intervals (1–X days ahead) to the figure caption.

The forecasts are narrowest for 1 day ahead and become more uncertain (i.e., broader) at longer horizons. This is why the credible intervals are disjoint — at each gap (representing the end of one forecast and the origin of another), the credible intervals on the left of the gap are 7, 14, or 28 days into the future, while the credible intervals on the right of the gap are only 1 day into the future.

5. Table 4: in the caption I would write out CRPS again. I am also struggling to interpret Table 4 without the raw CRPS values. It also seems, from the text, that the improvement is from comparing to the "4 weeks"? Can the authors make this clearer in the caption please.

We have revised this table, and now present mean log-transformed CRPS values by lead time (how far into the future we're predicting) rather than showing improvements as percentages. In each jurisdiction, the CRPS values are smallest for the shortest lead time (1–7 days ahead) and become larger as the lead time increases. These values can be interpreted as a probabilistic version of relative error, and we identify this in the table caption.

6. On Line 186, why do forecasts improve for "Omicron BA.2/BA.5" and "Multiple variants"?

After the Omicron BA.1 wave, we had quantitative data about temporal trends in test-seeking behaviour (which allowed teams to update mechanistic models in a way that wasn't possible prior to the Omicron BA.1 wave) and the rates of growth and decline in cases were slower than during the Omicron BA.1 wave. As we describe in further detail in a response to this reviewer's final comment (see below), the Omicron BA.1 wave was unprecedented in terms of diagnostics, (lack of) mobility restrictions, and other changes that we could only quantify in retrospect.

We have added the following text to the results (§3.2, Summary of forecast performance):

> This was likely due to a combination of having quantitative data about trends in test-seeking behaviour [34] (to inform the mechanistic models) and the slower rates of growth and decline in cases, relative to the "Omicron BA.1" period.

7. Figure 7: how is the score for each model measured against the ensemble? What does the dashed line represent? I don't see how this Figure shows that no model consistently outperformed the ensemble.

We now explain how to interpret skill scores in the revised methods section (§2.5, Forecast performance). We have added the following sentence to the figure caption (and moved the dashed line to indicate a skill score of zero):

> Positive values indicate performing better than the ensemble (with a maximum skill of 1 for a perfect point forecast), negative values indicate performing worse than the ensemble, and the horizontal dashed line marks the threshold skill of zero (performance equal to the ensemble).

And we have updated an associated sentence in the results section:

> Skill scores for each model, relative to the ensemble, were consistently below zero, further reinforcing the finding that no model consistently out-performed the ensemble (Figure 7).

8. As much as I enjoyed reading Section 3.9, I don't see what it actually brings to the manuscript? (Particularly since a lot of it can be attributed to [35])

While some of this was discussed at a conceptual level in [35] (a perspective article that broadly describes how mathematical modelling and data analyses supported COVID-19 policy responses in Australia and New Zealand), that paper covers none of the specific findings or visualisations that we present in Section 3.9. We have included this section because (as we now highlight in the introduction) the effective communication of forecast outputs to stakeholders was a critical aspect of our study, without which we would not have met our contractual obligations with the Australian Government.

We have revised the introduction to highlight the contributions presented in this manuscript, and have included the following text to explain why Section 3.9 is worth including:

> Because we were directly funded by the Australian Government to support Australia's public health responses, clear and effective communication of forecast outputs to stakeholders was of paramount concern, and we reflect on how we refined our reporting and visualisation over the study period.

9. Line 415: this reads like the authors were also trying to predict government policy and its impact, and that the ensemble failed to do so – this failure should be completed expected since the models would not be able to do this either and it would be unreasonable to expect them (and the ensemble) to do so. I think the sentence 413 to 415 is not needed at all and negatively changes the narrative.

We were not trying to predict policy, and did not intend to convey that impression. Rather, our aim was to highlight that this was a period where prediction would be particularly hard, and that our ensemble performed worst at a time when it was entirely reasonable to expect the models to perform poorly. Note that we were aware of these policy changes in advance (some of which were informed by other modelling studies conducted by our consortium, such as Ryan et al., Epidemics, 2024).

We have removed “a transition that was inherently challenging to predict” from the relevant sentence to avoid this confusion, and feel that the revised sentence remains relevant and worth keeping:

> The only period where the forecast did not capture the case data was the rapid emergence and decline of Omicron BA.1, which occurred as Australia pivoted from pursuing strong suppression to reopening, accepting substantial levels of local transmission, and rapid antigen tests were approved for self-testing and reporting [48].

Reviewer #3

Overall, this was a very interesting summary of the impactful work conducted in Australia during the COVID-19 pandemic. I think it would be of interest to the PLOS CB audience. At times, the paper felt like more of a report than a research article, and I encourage the authors to consider adding some more mathematical context for the models mentioned.

1. In some places, it isn't clear that the consortium reporting to the government included the authors of this work. I encourage the authors to go back through the abstract and introduction and make sure it is clear that they were a part of this consortium. Alternatively, if they were not, then I would make this distinction clearer.

   We thank the reviewer for this suggestion. We have updated both the abstract and the introduction to highlight that the authors were the very same consortium members who produced the forecasts:

   > As members of this consortium, who produced these forecasts at each week, we now retrospectively evaluate . . .
   >
   > Here we present an analysis of the ensemble forecasts of COVID-19 cases that were produced by the authors, under contract with, and reported weekly to, the Australian Government.

2. Could some more explicit details be given for the reader around the models, and data integration. I understand this might be challenging given the evolving nature of the models and the amount of data used, but I think it would be helpful for the reader to understand the types of models better so that we can understand their comparison.

   We now summarise the range of data sources that each team used during the study period in the methods section (§2.2, Forecasting teams and models):

   > Teams were able to use other data sources, in addition to the Australian COVID-19 case data. The MU team used global COVID-19 case data from Johns Hopkins [33] to inform their global autoregression model. The DST and UoA teams made use of Google mobility data to inform transmission estimates, the DST and UoM teams made use of behavioural survey data to inform case ascertainment [34], and the DST, UoA, and UOM teams made use of vaccination data from the Australian Immunisation Registry to inform vaccine coverage in their models. Where multiple teams used a common data source, they did not necessarily do so in an identical manner. For example, the DST and UoM teams made different assumptions about how much of the variation in case numbers reported by day-of-week was attributed to transmission versus case ascertainment.
