# Supplementary material for: Ensemble forecasts of COVID-19 activity to support Australia’s pandemic response: 2020–22
Source: PLoS Comput Biol. 2026 Apr 22;22(4):e1014199. doi: 10.1371/journal.pcbi.1014199 (PMC13124057; doi:10.1371/journal.pcbi.1014199)
Supplement: Attachment — Submitted filename: COVID19_Forecast_Review.docx [file pcbi.1014199.s002.docx]

# Review: *Ensemble forecasts of COVID-19 activity to support Australia’s pandemic response: 2020–22*

This manuscript presents an overview and evaluation of the ensemble forecasting effort that supported Australia’s COVID-19 response between 2020 and 2022. Several aspects of the presentation and methodological exposition require improvement before publication.

**More formal presentation**
Much of the description of the ensemble construction and evaluation is expressed verbally rather than formally. The reader is left with only a broad qualitative sense of how the ensemble was produced rather than a rigorous definition that would allow replication or scrutiny.
This reviewer strongly recommends formalizing the ensemble description with clear notation and explicit equations. For example, define the ensemble forecast X as a sample over contributing model distributions $\varphi_{i}$, i.e.:

$$p=\sum_{i} w_{i}\varphi_{i}(t),$$

$$with the sampling procedure clearly explained.$$

As is, statements such as “weights were allocated to each team rather than each model iteration” are extremely vague. It should be clear what the random variable represents, over what the averaging occurs, and how dependencies among trajectories are handled.

Without formalization, it is difficult to assess the ensemble’s statistical coherence. This reviewer would understand an the informal presentation were the article aimed at a policy-oriented audience, but this is a computational journal – formalism is not only acceptable, it is expected.

**Evaluation metrics**
The notion of “coverage” of credible intervals is used repeatedly but without a formal definition of what constitutes a credible interval in this context.
This reviewer suggests clearly supplementing the probabilistic measures (CRPS, bias, coverage) with more straightforward metrics, like absolute or relative case-count discrepancy, mean absolute error, or median absolute deviation. Such data should be presented both graphically and numerically

**Model identity and comparability over time**
The manuscript explicitly notes that individual modeling teams (UoA, UoM, DST, etc) updated or substantially revised their models during the study period, and that inclusion decisions were based on weekly peer review. Given these changes, it seems questionable to treat “UoA” or “UoM” as single, stable models during the ranking analyses (Fig. 6-7).
If a team’s model underwent structural revisions that materially altered its behavior, those forecasts effectively derive from different models. Treating them as one entity may confound the interpretation of the ranking and bias analyses. The authors should clarify whether their results aggregate across fundamentally distinct model versions and, if so, discuss the implications. It may be more defensible to define “model iteration” as the unit of analysis.

**Additional concerns and recommendations**

- The discussion of peer review and human judgment in excluding “suspect” models is interesting but could benefit from a more rigorous treatment of how this subjective step affects ensemble uncertainty. Is there a quantifiable impact of exclusion decisions on forecast variance or calibration?
- The discussion attributes reduced accuracy during Omicron BA.1 largely to epidemiological volatility and data limitations. While plausible, some quantitative decomposition (e.g., sensitivity to case ascertainment assumptions) would make this more convincing.
- The manuscript occasionally uses “forecast performance,” “bias,” and “skill” interchangeably. These should be defined explicitly, as readers from outside infectious disease modeling may not share the same intuitive understanding.

This reviewer would like to commend the authors for this work, and looks forward to reading an improved version in the near future.
